# Shaping the Microglia in Retinal Degenerative Diseases Using Stem Cell Therapy: Practice and Prospects

**DOI:** 10.3389/fcell.2021.741368

**Published:** 2021-12-13

**Authors:** Ni Jin, Weiwei Sha, Lixiong Gao

**Affiliations:** ^1^ Senior Department of Ophthalmology, The Third Medical Center of Chinese People’s Liberation Army General Hospital, Beijing, China; ^2^ Department of Endocrinology, The Second Medical Center and National Clinical Research Center for Geriatric Diseases, Chinese People’s Liberation Army General Hospital, Beijing, China

**Keywords:** microglia, stem cell therapy, retinal degenerative disease, microglial polarization, retinal inflammation

## Abstract

Retinal degenerative disease (RDD) refers to a group of diseases with retinal degeneration that cause vision loss and affect people’s daily lives. Various therapies have been proposed, among which stem cell therapy (SCT) holds great promise for the treatment of RDDs. Microglia are immune cells in the retina that have two activation phenotypes, namely, pro-inflammatory M1 and anti-inflammatory M2 phenotypes. These cells play an important role in the pathological progression of RDDs, especially in terms of retinal inflammation. Recent studies have extensively investigated the therapeutic potential of stem cell therapy in treating RDDs, including the immunomodulatory effects targeting microglia. In this review, we substantially summarized the characteristics of RDDs and microglia, discussed the microglial changes and phenotypic transformation of M1 microglia to M2 microglia after SCT, and proposed future directions for SCT in treating RDDs.

## 1 Introduction

The retina is a stratiform sensory tissue that consists of various cell types, including retinal pigment epithelium (RPE) cells, photoreceptors, intermediate neurons, retinal ganglion cells (RGCs) and glial cells (Malhotra et al., 2011; Madeira et al., 2015). Three distinct glial cell types are present within the retina: Müller cells, astrocytes, and microglia (Vecino et al., 2016). Müller cells are responsible for providing metabolic support to retinal neurons and regulating synaptic activity (Reichenbach and Bringmann, 2013). Together with Müller cells, astrocytes integrate the vascular and neuronal activity of the retina (Kolb, 1995). Microglia, the third type of retinal glial cell, are regarded as resident tissue macrophages and play important roles in retinal homeostasis (Langmann, 2007). Generally, microglia are proposed to originate from the yolk sac and are distributed widely in the whole retina. The main functions of microglia are phagocytosis and regulation of tissue inflammation. Two phenotypes of microglia have been identified: M1 microglia and M2 microglia. The former phenotype is generally considered pro-inflammatory, while the latter phenotype is anti-inflammatory (Tang and Le, 2016; Jiang et al., 2020).

Retinal degenerative diseases (RDDs) are a group of irreversible diseases characterized by the progressive degeneration of retinal cells, which eventually culminate in cell death. Certain conditions lead to an imbalance in the retinal microenvironment, which in turn causes retinal degeneration (Gorbatyuk and Gorbatyuk, 2013). The chronic inflammatory response is a nonnegligible part, where microglia serve as the culprit. Different therapeutic approaches have focused on controlling the activity of microglia to inhibit retinal inflammation (Karali et al., 2020; Lew et al., 2020), including stem cell therapy (SCT). The “control” was aimed at alleviating the functions of activated microglia, which normally refer to M1 microglia. However, recent studies also confirmed that SCT was likely to modulate microglial polarization toward the anti-inflammatory M2 phenotype (Jha et al., 2018). This review therefore offers a comprehensive overview of the interrelationship among RDDs, microglial modulation, and SCT. In addition, assumptions that SCT may be better used to treat RDDs by targeting microglial polarization are also discussed.

## 2 Characteristics of Retinal Degenerative Diseases

RDDs are a common form of neural degenerative diseases worldwide. They affect approximately 3.4 million people in the United States alone and are considered the dominant health issue. The number of patients continues to increase due to the aging of the population in industrialized countries. People with RDDs may suffer from a substantial loss of quality of life when their vision decreases to a certain extent (Wert et al., 2014). Additionally, RDDs have become a heavy burden for patients and society (Brown et al., 2006; Sapieha et al., 2010). For instance, over 250 billion dollars per year are spent on care for patients with age-related macular degeneration (AMD) in the United States (DeAngelis et al., 2017). A group of retinal diseases, such as retinitis pigmentosa (RP), diabetic retinopathy (DR), AMD, glaucoma, and Alzheimer’s disease (AD)-related retinal degeneration, are collectively known as RDDs (Yang et al., 2013; Madeira et al., 2015; Jin et al., 2019; Nashine et al., 2019). As substantial genetic and allelic heterogeneity exist among different RDDs, the specific classification of these diseases can be ambiguous (Wert et al., 2014). Considering the genetic perspectives, RDDs are classified as inherited retinal degeneration and noninherited retinal degeneration. For example, RP belongs to the former and DR to the latter. Currently, a cure for RDDs is unavailable. However, various therapies have been proposed, including pharmacotherapy (e.g., 9-cis-retinyl acetate), neuroprotection (application of neurotrophic factors), gene replacement (e.g., RPE65), retinal prostheses (restore visual function with devices), and SCT (Cideciyan et al., 2008; Zrenner et al., 2011; Trifunovic et al., 2012; Scholl et al., 2015; da Cruz et al., 2018). Although all current therapies have limitations in controlling the progression of RDDs, SCT is still one of the promising treatments. The implementation and related benefits of SCT will be discussed in the next sections.

The pathological characteristics of RDDs are similar but different. For example, loss of retinal neurons occurs in all RDDs, while neovascularization is a unique feature of AMD and DR. More importantly, a certain RDD itself can also be highly variable. Patients with RP can develop symptomatic visual loss both in childhood and in middle age (Hamel, 2006). Many patients suffer from nyctalopia in adolescence and loss of the mid-peripheral visual field in early adulthood (Hartong et al., 2006). The corresponding pathogenic process in RP is the gradual degeneration of two photoreceptor cells: the primary atrophy of rod cells and the subsequent death of cone cells (Wert et al., 2014). DR is a common and specific microvascular complication of diabetes. In the early stages, DR is largely asymptomatic. However, it can result in retinal detachment and sudden loss of vision as the disease progresses (Lechner et al., 2017). Chronic exposure to hyperglycemia and other risk factors (e.g., hypertension) is postulated to enhance the biochemical and physiological changes that lead to microvascular damage of the retina (Cheung et al., 2010). Vasculopathy subsequently leads to retinal hypoxia and harmful neovascularization (Grossniklaus et al., 2010). Besides, DR exhibits characteristics of low-grade chronic inflammation. Increased expression of inflammatory cytokines, such as TNF and IL-1β, subsequently increases the endothelial cell permeability, promotes the breakdown of blood-retinal barrier (BRB), and induces the adhesion of leukocytes (Madeira et al., 2015). Affecting the macula, AMD compromises the central, fine vision of patients. It has become the leading cause of visual impairment in the aging population, especially in those over 55  years of age (DeAngelis et al., 2017). Two major forms of this disease have been identified. “Dry AMD” is the most prevalent form related to slow progressive degeneration of the RPE and loss of photoreceptors. “Wet AMD” is the less frequent but more symptomatic form characterized by the formation of choroidal neovascularization (CNV). Similar to harmful neovascularization in DR, CNV causes intraretinal or subretinal leakage, hemorrhage, and RPE detachment (Salvi et al., 2006; Velez-Montoya et al., 2013). Macula-affected CNV is the primary cause of vision loss in patients with wet AMD (Ambati and Fowler, 2012). The two AMD forms are not mutually exclusive, as one patient can present both pathological changes (Ashraf and Souka, 2017). Glaucoma is often divided into two major subtypes, open angle and angle closure. Open-angle glaucoma is a chronic process. Patients are often asymptomatic until vision loss has progressed significantly. Angle-closure glaucoma can be an acute process with more immediate symptoms and tends to be more destructive (Mantravadi and Vadhar, 2015). Both subtypes have typical structural and functional defects characterized by the death of a substantial number of RGCs in the inner retina and the loss of their axons in the optic nerve (Quigley, 2011). The loss of RGCs in patients with glaucoma is closely related to the level of intraocular pressure (Weinreb et al., 2014). AD also causes retinal degeneration, which becomes a prominent feature of AD pathology (Koronyo-Hamaoui et al., 2011; Mirzaei et al., 2020). AD patients may suffer from various visual impairments such as loss of contrast and color sensitivity, limited visual field, compromised visual attention and reduced stereopsis (Hart et al., 2016). The hallmark pathology in ocular tissues of AD patients is the deposition of amyloid β (Aβ) and phosphorylated tau protein aggregates, which lead to the RGC degeneration and thinning of retinal nerve fiber layer (Gao et al., 2015; Ashok et al., 2020).

In addition to the abovementioned differences, chronic inflammatory responses also play important roles in the development of RDDs (Madeira et al., 2015). A group of main immune cells within the central nervous system (CNS) and the retina, namely, microglia, plays major roles in chronic inflammation (Li et al., 2015; Rashid et al., 2019). Damage to retinal cells activates microglia to restrict injuries and eliminate cellular debris. However, the overactivation of microglia results in the excessive production of inflammatory factors, which damages retinal cells and aggravates other harmful processes, such as enhancing Aβ-induced toxicity (Qin et al., 2002; Madeira et al., 2015). Therefore, microglia play leading roles in the initiation and persistence of inflammation within RDDs, which subsequently traps RDDs into vicious cycles. More detailed descriptions of the conditions are provided below.

## 3 Microglia and the Retina

### 3.1 Origin, Maintenance, and Morphology of Microglia

Microglia were first declared a population in the CNS different from neurons and astrocytes by del Río-Hortega (1993). Previously, microglia in the brain were presumed to have a hemopoietic origin, with monocytes serving as their precursor cells (Imamoto and Leblond, 1978). In contrast, an authoritative study reported that microglia were mainly derived from primitive macrophages in the yolk sac (Ginhoux et al., 2010). Hoeffel et al. (2015) used a fate mapping system to reveal two waves of erythromyeloid precursors (EMPs) in the yolk sac of mice. The first wave of E7.5 progenitors gave rise to early EMPs and subsequently differentiated into primitive macrophages. The second wave of EMPs generated other hematopoietic progenitors and differentiated into hematopoietic stem cells (HSCs) that colonize the fetal liver. Between these two waves, the first wave of EMPs is the origin wave for microglia (Hoeffel et al., 2015). Consistent with these findings, another *in vitro* study confirmed that the vast majority of mouse microglia in the brain originated from EMPs distinct from HSCs (Gomez Perdiguero et al., 2015). A study of human tissue indicated that microglia migrated to the retina mainly from two sources: The retinal margin and the optic disc (Diaz-Araya et al., 1995). In summary, microglia are generally considered to originate from the yolk sac and invade the retina later. Afterwards, the production of microglia is different from the process occurring in the developmental period. Although the origin of microglia in vertebrate model animals is clear, the ontogeny of human microglia is still a matter of debate due to the lack of direct evidence.

Microglia exhibit a self-renewal pattern under both physiological and pathological conditions (Bruttger et al., 2015). After comparing cases, the density of microglia was shown to be remarkably stable in young and aged brains of both mice and humans in one study. Coupled proliferation and apoptosis maintained the turnover of microglia, while no extra infiltration of monocytes was involved. Additionally, an average of 0.69% of microglia were in S phase at a particular time, which allowed researchers to estimate that the microglia population in the mouse brain is renewed every ∼95 days (Askew et al., 2017). Another study used a special strategy to retrospectively analyze the birth date of microglia isolated from human adults and found that the age of microglia can be long to 6 decades. The majority of microglia in the healthy human cortex were replaced by newly produced cells at a median rate of 28% per year (or 0.08% per day) and the average age was 4.2 years (Reu et al., 2017). These results established that the microglial population in the human brain is sustained by continuous slow turnover throughout life. However, researchers have not clearly determined whether microglia in the retina have the same self-renewal pattern. In addition to self-proliferation, bone marrow (BM)-derived macrophages and monocytes invade the CNS and contribute to the microglial pool under specific conditions, such as irradiation (Jin et al., 2017).

Normally, microglia adopt a quiescent phenotype characterized by very small stomata and extensively ramified filopodia-like processes. They monitor the entire CNS, including the retina, by continuously moving their processes (Karlstetter et al., 2015). Microglia in the CNS are activated when encountering acute damage and adopt an amoeboid shape. Amoeboid microglia differ from ramified microglia with spherical shapes because they lack processes and have numerous phagocytic vacuoles (Boche et al., 2013). The label “amoeboid” implies that such cells are capable of motility. In addition, several other morphological states of microglia have been identified, such as rod cells, multinucleated cells, and “dystrophic” microglia. Rod cells have elongated nuclei, scant cytoplasm, few processes and are most notable in chronic disorders. Multinucleated cells form as a reaction to indigestible material and are commonly observed in mycobacterial infection. “Dystrophic” microglia are cells with dysfunction due to aging (Boche et al., 2013).

### 3.2 Microglial Polarization Toward Different Phenotypes

Activated macrophages have consistently been shown to present different phenotypes in several inflammation-induced human diseases (Shapouri-Moghaddam et al., 2018). Microglia are recognized as a specialized macrophage population within the CNS (McMenamin et al., 2019). Similar to macrophages, microglia have two activation phenotypes, the M1 phenotype and the M2 phenotype (Tang and Le, 2016), which represent simplified models to describe two polar states of inflammatory responses, namely, pro- and anti-inflammatory responses. Polarization refers to the activation of microglia toward a specific phenotype (Kobashi et al., 2020).

Classical M1 microglia contribute to the release of pro-inflammatory substances such as TNF-α, IL-1β, IL-6, superoxide, inducible nitric oxide synthase (iNOS), reactive oxygen species, and proteases (Le et al., 2001; Indaram et al., 2015; Collmann et al., 2019). These substances promote neuroinflammation and result in a poor outcome. For example, the secretion of pro-inflammatory molecules such as TNF-α and IL-1β by microglia cause chronic inflammation and lead to the damage of the BRB in the diabetic retina (Kinuthia et al., 2020). Several markers, including CD11b, CD16, CD32, CD68, and CD86, are used to identify M1 microglia (Jiang et al., 2020). This phenotype is typically induced by stimuli such as lipopolysaccharide (LPS) or granulocyte-macrophage colony-stimulating factor (GM-CSF) (Liu et al., 2019; Kobashi et al., 2020). In contrast, M2 microglia dampen the inflammatory response by producing four major anti-inflammatory cytokines: IL-4, IL-13, IL-10, and TGF-β (Butovsky et al., 2005; Zhou et al., 2012). For example, IL-4 has been shown to decrease the production of several pro-inflammatory mediators, and IL-10 inhibits the activity of many pro-inflammatory factors (Vanderwall and Milligan, 2019). In addition, these cytokines promote the release of neurotrophic factors such as insulin-like growth factor 1 (IGF-1) to increase neuron survival (Suh et al., 2013). M2 microglia also play a beneficial role in CNS diseases. By secreting chitinase-3-like protein 3, IL-10, and TGF-β, M2 microglia promote angiogenesis and ultimately mitigate blood-brain barrier (BBB) leakage (Zhu et al., 2019). In addition, M2 microglia-derived exosomes attenuate ischemic brain injury and promote neuronal survival (Song et al., 2019). M2 microglia can be identified by markers such as CD206, CCL22 and arginase-1 (Arg-1) (Jiang et al., 2020), and can be induced by IL-4 (Kobashi et al., 2020).

### 3.3 Roles of Microglia in the Normal Retina

#### 3.3.1 Microglia Play a Supporting Role in the Development of the Retina

During the developmental period, the proliferation, survival, and neurite outgrowth of embryonic neurons are promoted by microglia-mediated trophic factors (Morgan et al., 2004). More recently, microglia have been shown to play an important role in the postnatal maturation of retinal photoreceptors (Jobling et al., 2018). They also participate in the retention of selected neurons and the elimination of unwanted cells, a process that is achieved by the microglia-mediated phagocytosis of cellular debris, pruning of weak presynaptic terminals of RGCs, and decrease in costly neural connections deemed to be unfit for proper function (Bodeutsch and Thanos, 2000; Schafer et al., 2012). In addition, microglia are important in the process of retinal vascularization, which comprises two phases. The first phase is that hyaloid vessels extend from the optic disk to the lens and supply blood and nutrients to the developing eye. In the second phase, hyaloid vessels regress and the retina develops its own independent vascular network (Li et al., 2019a). Microglia-mediated apoptosis of vascular endothelial cells contributes to the main step in the first phase (Lobov et al., 2005), the failure of which can cause persistent hyperplastic primary vitreous in the postnatal period. In the next phase, microglia are closely apposed to endothelial tip cell filopodia, which guide blood vessel growth through the tissue (Checchin et al., 2006).

#### 3.3.2 Microglia Keep Silent and Supervise the Healthy Retina

After development, microglia maintain the ramified morphology with small cell bodies and long cellular protrusions and form a non-overlapping microglia network, which provides a comprehensive surveillance coverage of the entire retina (Damani et al., 2011). Some researchers propose that there are mechanisms of inhibiting microglial activation in the healthy retina to prevent deleterious effects. Cell types including photoreceptors, vascular endothelial cells, ganglion cells, and Müller cells are involved in this process (Dick et al., 2003; Langmann, 2007). Especially, microglia perform an active cross-talk with Müller cells. On the one hand, Microglia can directly trigger the release of several neurotrophic factors from Müller cells. On the other hand, Müller cells can limit microglial reactivity and potentially transform activated microglia into their ramified surveillance state (Karlstetter et al., 2015). However, these mechanisms lose control of microglial behavior in pathological conditions where microglia become activated (Rathnasamy et al., 2019).

### 3.4 Roles of Microglia in Retinal Degenerative Diseases

#### 3.4.1 Microglia Phagocytose Both Wastes and Living Cells

Phagocytosis by microglia has been extensively studied in adult individuals. The main phagocytic targets of microglia in the brain and retina include pathogens, dead cells, dying cells and protein aggregates (McMenamin et al., 2019). Following interventions such as axotomy, microglia that removed debris of neuronal cells in the postnatal retina were reactivated later in life to phagocytose damaged neurons (Thanos, 1991). Nonetheless, phagocytosis by microglia is a double-edged sword. In a mouse model of RP, activated microglia phagocytosed not only cell debris (Gupta et al., 2003) but also living neurons and accelerated retinal lesions (Zhao et al., 2015). In addition, microglia contributed to the leakage of the BRB by phagocytosing endothelial cells in another rat model of DR (Xie et al., 2021). The molecular mechanisms involved in microglial phagocytosis are still under investigation. Retinal microglia promote the clearance of infectious microbes by expressing receptors such as Toll-like receptors (TLRs) and dectin-1 (Maneu et al., 2011; Kochan et al., 2012). The expression of TLR4 in microglia contributes to their activation and phagocytosis of photoreceptor proteins (Kohno et al., 2013). In addition, phagocytosis is mediated by triggering receptor expressed on myeloid cells 2 (TREM2) and Mer receptor tyrosine kinase (MerTK) in the brain (Neher et al., 2013; Kim et al., 2017). However, whether they participate in microglial phagocytosis of apoptotic retinal neurons is still unknown.

#### 3.4.2 Microglia Promote the Neovascularization

As mentioned above, microglia play an important role in retinal vascularization during development. It is noted that microglia also contribute to the retinal neovascularization in retinal diseases, which is associated with the microglia migration and microglia-related inflammation (Usui-Ouchi et al., 2020). In AMD, microglia contribute to the formation of CNV through accumulating in the subretinal space, releasing inflammatory cytokines (IL-1β, TNF-α, IL-6 and TGF-β), producing pro-angiogenic cytokines and growth factors (VEGF and PGF), and activating microglial VEGF receptors (VEGFR1 and VEGFR2) (Alves et al., 2020). In DR, angiogenesis and inflammation are not independent. Microglia might induce the neovascularization by releasing pro-angiogenic mediators, including cytokines, growth factors, and proteases (Altmann and Schmidt, 2018).

#### 3.4.3 Microglia Aggravate Retinal Inflammation

Regarding immune surveillance and regulatory functions, microglia act as surveillants with their processes continuously extending and retracting in all directions in a random and repeated manner. These cells can sense subtle changes in the retinal microenvironment through various surface receptors and rapidly react to these changes (Nimmerjahn et al., 2005). During the rapid phase of RDDs, microglia are activated immediately and proliferate and migrate to degenerative sites (Zhou et al., 2017). The morphology of microglia also changes to an amoeboid shape (Zhou et al., 2017). Simultaneously, by secreting TNF-α and IL-1β, microglia participate in retinal inflammation and function as a “double sword” again (Krady et al., 2005; Sivakumar et al., 2011). On the one hand, these cytokines initiate immune defenses. On the other hand, they aggravate the death of retinal neurons and damage the integrity of the BRB (Claudio et al., 1994; Tezel and Yang, 2004; Abcouwer et al., 2008). In addition, microglia release pro-inflammatory cytokines, including IL-3, IL-6, IL-8, IL-10, IL-12, and IL-18 (Liu et al., 2012; Grigsby et al., 2014). Moreover, microglia express major histocompatibility complex class II (MHC II) and share phenotypic characteristics with professional antigen-presenting cells (Penfold et al., 1993). Normally, quiescent microglia express low levels of MHC II proteins (Kreutzberg, 1996). Once activated, microglia upregulate MHC II molecules that are required for antigen presentation to T cells. This feature suggests the capability of microglia to reactivate primed T cells entering the CNS (Rawji and Yong, 2013). In addition, microglia are related to the activation of the complement system, an innate immune response that protects host tissue from immunological stimuli. Luo et al. identified microglia and RPE cells as the main sources of retinal complement gene expression (Luo et al., 2011). Unfortunately, in DR models, microglia contribute to the deposition of complement C3 and C1qa, which promote the apoptosis of photoreceptors and RGCs (Howell et al., 2011; Rutar et al., 2011).

It can be seen that microglia exert M1 phenotype due to the release of pro-inflammatory factors. However, some studies propose that M2 microglia also exist in the retina of RDDs. The polarization tendency of microglia in the retina is an intricate process, which is a topic of extensive debate. According to Arroba and Valverde (2017), M2 microglia and M1 microglia both appear at the early stage of DR; however, during the progression of DR, the M1 phenotype is maintained, whereas the M2 phenotype decreases. Different results were obtained for the polarization of microglia in a model of retinal degeneration. Initially, most of the activated microglia (CD86^+^, CD16/32^+^, CD40^+^) tended to exhibit the M1 phenotype and release pro-inflammatory cytokines. Although no M2 microglia (CD206^+^) were observed at this stage, many microglia were colabeled with CD86 and CD206, indicating an intermediate state of microglial polarization (Zhou et al., 2017). Another study examining oxygen-induced retinopathy models found that M1 microglia dominated during the initial period, but M2 microglial activity predominated during the late phase (Li et al., 2021a). Consistent with these findings, in a study of light-induced retinal damage, pro-inflammatory M1 macrophages/microglia dominated in the early phase, while the chronic postexposure period was accompanied by persistent upregulation of the M2 phenotype. The authors speculated that resident macrophages/microglia might switch to the M1 phenotype in response to light damage; however, infiltrated BM-derived macrophages/microglia mainly contributed to M2 polarization (Jiao et al., 2015). Therefore, more research is needed to better understand the spatiotemporal cadence of microglial polarization in the retina.

Taken together, in the diseased retina, microglia exert both beneficial and detrimental effects. However, the function of promoting inflammation is widely considered pernicious. Many treatments are currently focusing on the regulation of microglial behavior patterns. The question is whether all microglia should be eliminated. As the two phenotypes of microglia are closely linked to the progression of retinal inflammation, microglia polarization may be a vital target for the treatment of RDDs. Since SCT plays a beneficial role in treating retinal diseases, we propose that the modulation of microglia phenotype following SCT is also valuable, which will be discussed in the next sections.

## 4 Basic Approaches of Stem Cell Therapy to Develop Therapeutic Effects

**FIGURE 1:**
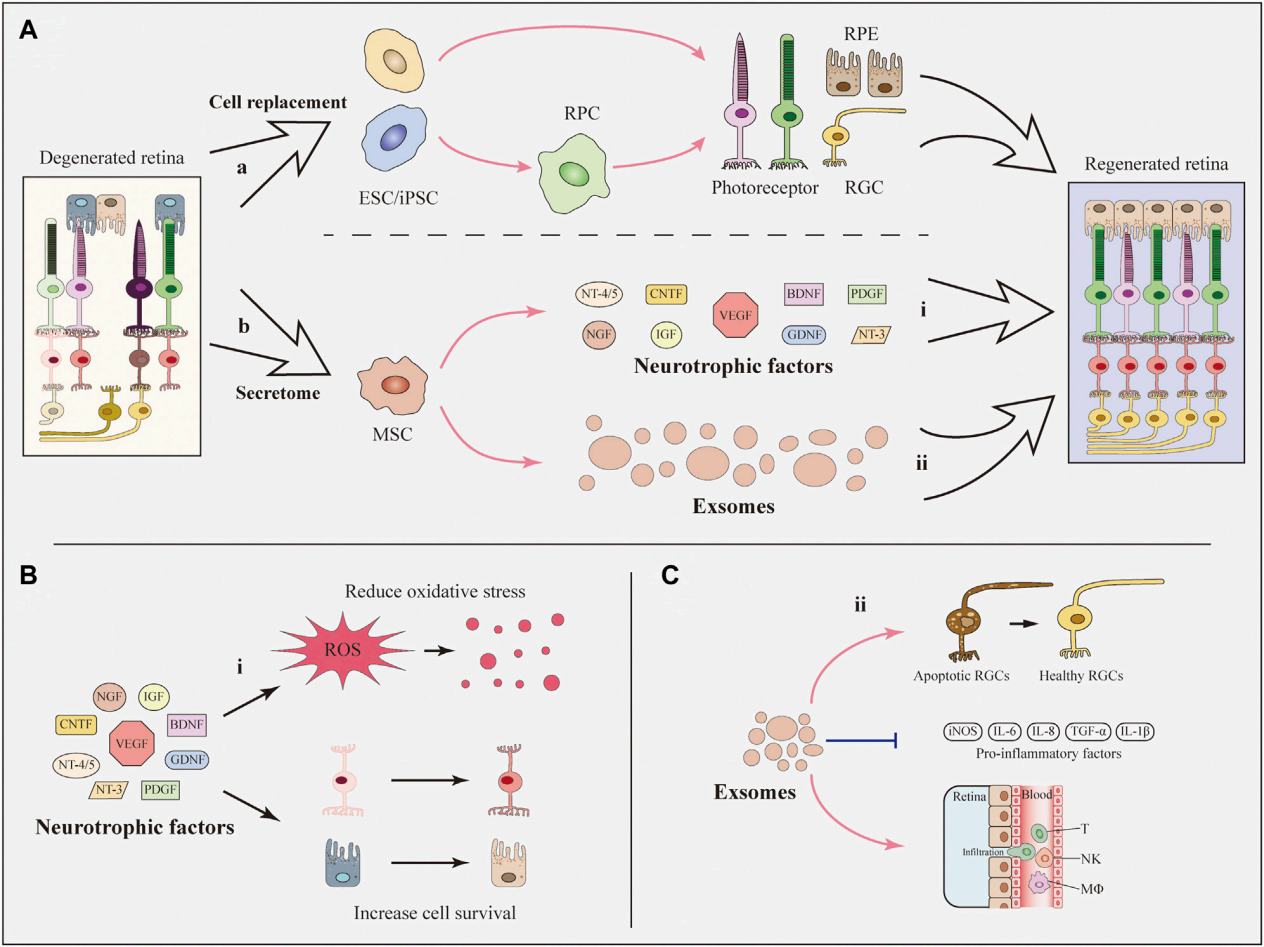
Basic approaches of SCT to develop therapeutic effects. **(A)** Basically, stem cells contribute to the retinal recovery through two approaches: Cell replacement and the secretome. **(i)** iPSCs, ESCs, and RPCs can differentiate into retinal cells including RGCs and photoreceptors and can be used for cell replacement. **(ii)** MSCs secret neurotrophic factors and exosomes which are beneficial for the recovery of regenerated retina. **(B)** Neurotrophic factors contribute to the reduction of oxidative stress and the survival of retinal cells. **(C)** MSC-derived exosomes prevent the decline of RGCs and reduce the expression of inflammatory factors as well as the infiltration of inflammatory cells.

Stem cells are a population of undifferentiated cells characterized by the ability to extensively proliferate and differentiate into different types of cells and tissues (Kolios and Moodley, 2013). Recent years, SCT has been extensively applied in the treatment of various diseases, including neurological disorders (Alessandrini et al., 2019), heart diseases (Muller et al., 2018), and discogenic back pain (Barakat et al., 2019). The eye is “immune privileged” due to the protection of the BBB and BRB (Forrester et al., 2018). In addition, the high operability and convenient observability make the eye an ideal target organ for stem cell transplantation. Currently, numerous studies have transplanted RPE cells, photoreceptors and RGCs derived from induced pluripotent stem cells (iPSCs), embryonic stem cells (ESCs) and retinal progenitor cells (RPCs), and some of them have advanced to clinical trials (Liu et al., 2017; Mandai et al., 2017; Zhang et al., 2020b). Previously, we used human ESCs (hESCs), human mesenchymal stem cells (MSCs), human RPCs (hRPCs) and neural stem cells (NSCs) to treat retinal degeneration in animal models (Qu et al., 2017; Zhai et al., 2020; Li et al., 2021b). Stem cells are directly transplanted or differentiated into anticipated cell types prior to transplantation. Basically, stem cells contribute to the retinal recovery through two approaches: cell replacement and secretome (Figure 1). Here, we will briefly discuss these two approaches.

### 4.1 Cell Replacement

To date, SCT has been widely studied in animal models for cell replacement in RDDs. ESCs and iPSCs have the greatest potential for cell replacement in RDDs while MSCs and NSCs are sparsely reported to differentiate into retinal cells after transplantation (Mead et al., 2015). As the loss of RPE cells and photoreceptors primarily contributes to visual impairments in these RDDs, studies have more frequently focused on replacing these two cell types.

RPE cells are a layer of pigment cells that transport nutrients from the blood to photoreceptors and digest deciduous disks from the outer segment of photoreceptors (Ma et al., 2019). Both hESCs and human iPSCs (hiPSCs) can be induced to generate RPE cells (Figure 1) (Klimanskaya et al., 2004; Buchholz et al., 2009). In 2018, successful delivery of hESC-derived RPE cells in two patients was reported in a clinical trial and indicated the feasibility and safety of hESC-derived RPE transplantation (da Cruz et al., 2018). Additionally, clinical trials of cell replacement based on RPCs and iPSCs have been conducted in recent years (Liu et al., 2017; Mandai et al., 2017). A scalable protocol was published that facilitated the production of high-quality RPE cells in a short time span and convenient application for both investigative and clinical use to solve the problem of high cost and low purity when generating RPE cells. Pure functional RPE monolayers have been derived from hiPSCs within 90 days using simplified 2D cultures (Buchholz et al., 2013; Maruotti et al., 2015; Michelet et al., 2020). However, no further studies have adopted this protocol to date.

Photoreceptors are irreplaceable in sensing light signals and visual cues by converting exogenous cues into bioelectrical signals (Gollisch and Meister, 2010). Photoreceptors have been generated from hESCs, iPSCs and RPCs (Figure 1) (Qiu et al., 2007; Lamba et al., 2009; Lamba et al., 2010). The replacement of photoreceptors differentiated from iPSCs has been studied in a mouse model. Human iPSCs differentiated into photoreceptors and subsequently survived and integrated into the host retina (Lamba et al., 2010). Recently, hRPCs transplanted in patients with RP successfully differentiated into photoreceptors in a clinical trial. The results showed the long-term safety and feasibility of vision repair following hRPC transplantation (Liu et al., 2017). One important obstacle to the clinical use of photoreceptors is that an appropriate source of precursor cells is required (Schmeer et al., 2012). Lately, an optimized protocol for generating labeled and transplantable photoreceptor precursors from hESCs was published, which was an advance for future research (Markus et al., 2019).

Although cell replacement therapy has been implemented in many animal studies, limitations still confine its further applications in clinical trials due to several unsolved challenges. The low survival rate of transplanted cells and potential tumorigenicity risks have been widely studied (Ballios et al., 2015; Wang et al., 2020). Moreover, the immune rejection of allografts (Baker and Brown, 2009), which is partially associated with the pro-inflammatory stimuli caused by M1 microglia, also must be resolved in future studies.

### 4.2 Secretome

The secretome of stem cells is defined as the set of molecules and biological factors secreted by stem cells into the extracellular space by mechanisms including protein translocation, exocytosis, and vesicle or exosome encapsulation (Xia et al., 2019; Daneshmandi et al., 2020). Among these molecules and factors, neurotrophic factors and exosomes are widely reported to play beneficial roles in retinal diseases (Wen et al., 2012; Xiao and Le, 2016; Hu et al., 2017; Mead et al., 2018). Although paracrine effects have been discovered in many types of stem cells, including MSC, NSC, ESC, and iPSC (Khan et al., 2015; Bakondi et al., 2017; Mead et al., 2018; Taheri et al., 2019; Zhang et al., 2020a), MSC is the major force of secreting neurotrophic factors and exosomes (Mead et al., 2015; Mead and Tomarev, 2020). Thus, we will focus on the secretome of MSCs and related therapeutic effects in the following sections.

#### 4.2.1 Neurotrophic Factors

Neurotrophic factors are growth factors that nourish neurons and promote the survival and regeneration of neurons, including photoreceptors, RGCs and RPE cells (Wen et al., 2012; Xiao and Le, 2016). They are classified into three families based on their structures, receptors, and signaling pathways: 1) the neurotrophin family, including nerve growth factor (NGF), brain-derived growth factor (BDNF), neurotrophin-3 (NT-3), and NT-4/5; 2) the glial cell-derived neurotrophic factor (GDNF) family, including GDNF, neurturin, persephin, and artemin; and 3) neuropoietic cytokines, including ciliary neurotrophic factor (CNTF) and cardiotrophin-1 (Boyd and Gordon, 2003). Transplantation of stem cells can play positive roles of secreting neurotrophic factors which further improve the survival of retinal neurons.

MSCs secrete neurotrophic factors via a paracrine mechanism (Figure 1), which dampens retinal degeneration instead of replacing damaged cells (Bakondi et al., 2017). MSCs derived from different sources secret similar neurotrophic factors. To be specific, bone marrow derived MSCs (BMSCs) secret an array of neurotrophic factors involving all the three families including CNTF, BDNF, GDNF, platelet derived growth factor (PDGF), NGF, NT-3, NT-4/5 (Mead et al., 2015), IGF-1, basic fibroblast growth factor 2 (FGF2), pigment epithelium-derived factor, and erythropoietin (Usategui-Martin et al., 2020). Analogously, adipose derived MSCs (ADMSCs) contribute to the release of hepatocyte growth factor, CNTF, IGF (Fontanilla et al., 2015), FGF2, VEGF, NGF, BDNF, GDNF, NT-3, and PDGF (Mead et al., 2014; Ezquer et al., 2016).

Many studies have confirmed the protective effects of MSC-secreted neurotrophic factors on eye diseases (Figure 1). For example, in an experimental optic nerve crush model, intravitreal transplantation of BMSCs that secrete GDNF and BDNF resulted in a greater number of living RGCs than in the control group (Hu et al., 2017). In addition, intravitreal administration of murine ADMSCs prevented RGC loss and reduced oxidative stress in the retina with increasing levels of NGF, FGF2 and GDNF in a diabetic mouse model (Ezquer et al., 2016). As another option, neurotrophic factors have also been delivered through direct intravitreal injections (Daly et al., 2018). However, researchers have not clearly determined whether the paracrine effects of neurotrophic factors observed following SCT are more efficient than direct injection of neurotrophic factors. More research is required to expand the function of stem cell-secreted neurotrophic factors and explore their neuroprotective effects on retinal cells.

#### 4.2.2 Exosomes

Exosomes are cell-derived nanovesicles that have low toxicity, exquisite target-homing specificity and the potential for drug/gene delivery (Kalluri and LeBleu, 2020). The secretion of exosomes plays positive roles in SCT treating RDDs. Among several types of MSCs, adipose, BM and umbilical derived-MSCs are the main sources of secreted exosomes (Mead and Tomarev, 2020).

MSC-derived exosomes have many functions, such as neuroprotection and immunoregulation. In a mouse model of glaucoma, exosomes derived from BMSCs significantly reduced the number of degenerating axons in the optic nerve. Meanwhile, exosomes prevented the decrease in RGC function in the early phase (Figure 1) (Mead et al., 2018). Regarding immunoregulation, MSC-derived exosomes substantially suppressed the progression of autoimmune uveoretinitis in a rat model by reducing the infiltration of inflammatory cells, such as T cell subsets (Figure 1) (Bai et al., 2017), and alleviated the expression of inflammatory mediators in the injured retina, including TNF-α, monocyte chemoattractant protein-1 and intercellular adhesion molecule 1 (Harrell et al., 2018). However, the key component of exosomes remains unknown. More importantly, the production of highly purified exosomes with stable long-term functional efficacy for clinical trials is a great challenge (Zhang et al., 2021).

## 5 Immunomodulatory Effects of Stem Cell Therapy Targeting Microglia

SCT has been proven to possess broad immunomodulatory potential in neurodegenerative diseases by regulating inflammatory responses. As mentioned above, microglia play a leading role in the immune system of the retina through polarization toward two phenotypes. Here, we emphatically discuss the immunomodulatory effects of SCT on regulating the microglial polarization and microglia-mediated inflammation.

### 5.1 Stem Cell Therapy Inhibit M1 Microglial Polarization

In pathological states, microglia proliferate rapidly with a distinctly increased number of cells. At the same time, they migrate to degenerative sites upon activation. In many studies of RDDs, microglia present the M1 phenotype by releasing pro-inflammatory factors (Yuan and Neufeld, 2001; Krady et al., 2005; Zeng et al., 2005). To date, many stem cell treatments have been shown to exert regulatory effects on M1 microglia both *in vitro* and *in vivo*. By coculturing the retinas of adult rats with human BMSCs (hBMSCs) *in vitro*, researchers found that the number of CD68^+^ ameboid microglia decreased and the loss of RGCs was prevented (Teixeira-Pinheiro et al., 2020). Consistently, the activation of CD11b^+^ M1 microglia was inhibited by coculturing with MSC-derived microvesicles in an *in-vitro* model. Meanwhile, the production of pro-inflammatory molecules by M1 microglia, such as TNF-α, IL-6, IL-1β and iNOS, was reduced (Jaimes et al., 2017). In our previous *in-vivo* study, by transplanting organoid-derived hRPCs into the subretinal space of rat RDD models (RCS rats), we found that the number of Iba1^+^/CD68^+^ phagocytic M1 microglia was significantly lower in the transplantation group than that in the control group (Zou et al., 2019). In addition, we performed a combined transplantation of human MSCs (hMSCs) and hRPCs into the subretinal space of RCS rats. The number of Iba1^+^ retinal microglia was significantly reduced following transplantation, especially in the combined transplantation group. The expression levels of TNF-α and IL-1β were decreased while the expression levels of neurotrophic NGF and BDNF were increased (Qu et al., 2017).

**FIGURE 2:**
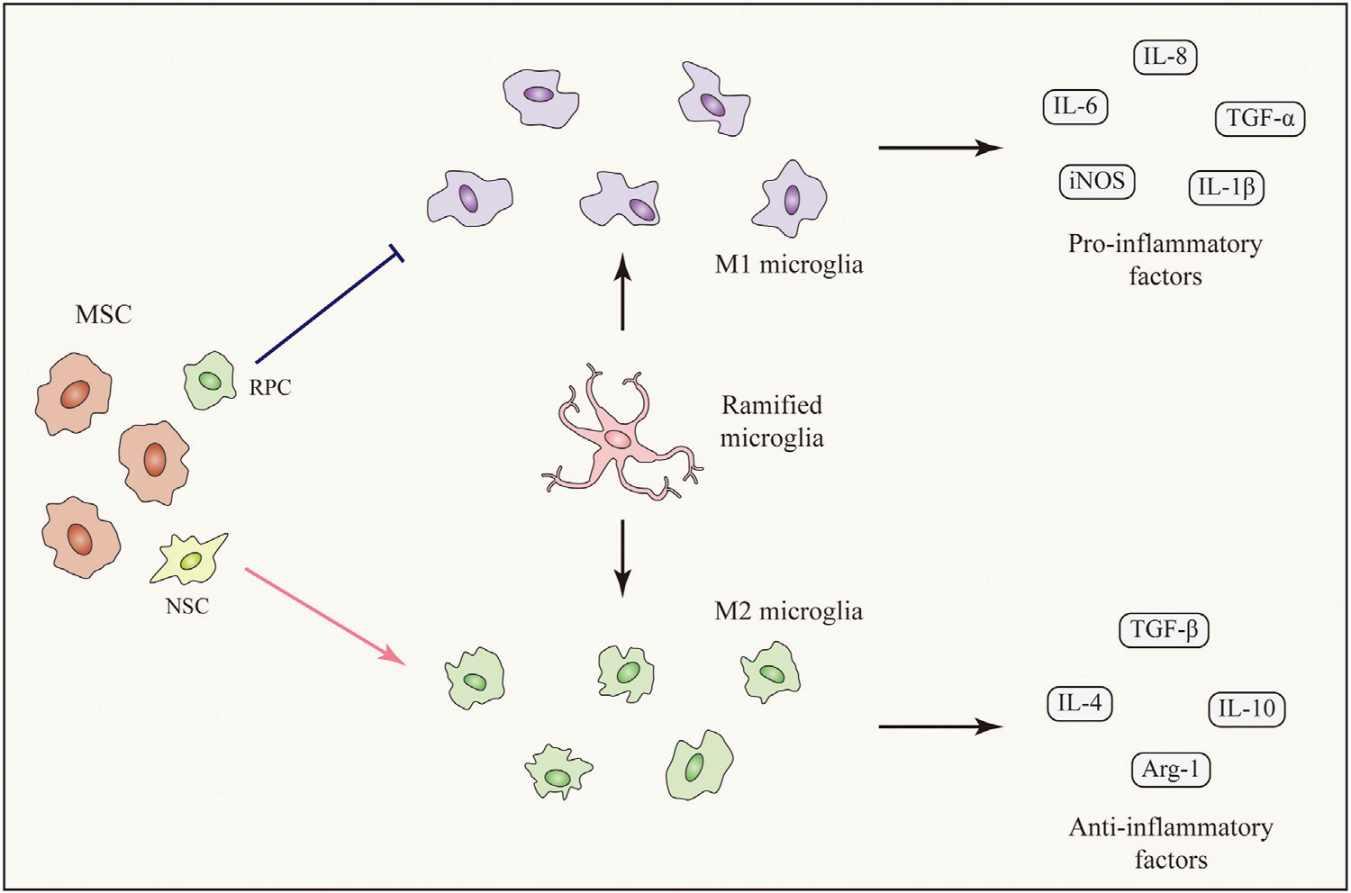
Immunomodulatory effects of SCT targeting microglia. Ramified microglia can be activated with two phenotypes, namely M1 phenotype and M2 phenotype. Stem cells including MSCs, RPCs, and NSCs can modulate microglial polarization (M1-M2), among which MSCs are the major force. MSCs inhibit M1 microglial polarization and decrease the expression level of pro-inflammatory factors. On the contrary, M2 microglial polarization is enhanced and the expression levels of anti-inflammatory factors are increased. The transition of microglial phenotype alleviates the inflammation and improves the living-conditions of neuronal cells.

All these results confirmed the inhibitory effects of SCT on M1 microglia (Figure 2). Along with the inhibition of M1 microglia activation, the expression levels of inflammatory factors and cytotoxic molecules were decreased, which can alleviate the chronic injury of the retina.

### 5.2 Stem Cell Therapy Enhance M2 Microglial Polarization

As mentioned above, polarization of microglia to the M2 phenotype is beneficial for treating RDDs. Several studies have shown that SCT can promote M2 microglial polarization and MSCs are the major force of this function (Zanier et al., 2014; Park et al., 2016; Jha et al., 2018). In an *in-vitro* study of LPS-stimulated microglia, treatment with ADMSCs or ADMSC-derived GDNF enhanced the expression of the M2 marker CD206. The expression levels of anti-inflammatory IL-10 and TGF-β were upregulated (Zhong et al., 2020b). As for *in-vivo* studies, the intravitreal injection of concentrated conditioned media from ADMSCs restored the M1-M2 balance in a mouse model of visual deficits following mild traumatic brain injury. This treatment increased the number of Arg-1^+^ M2 microglia along with the increased expression of anti-inflammatory cytokines. As a result, the inflammation-related loss of endothelial barrier integrity and retinal cells was suppressed (Jha et al., 2018). Unfortunately, the number of studies which discovered M2 polarization-promotion effect following SCT in the retina are limited. However, many studies focusing on brain injury have confirmed the effects of MSCs on enhancing M2 microglial polarization. In a mouse model of neurodegeneration, the intracerebral transplantation of MSCs promoted M2 polarization and increased the expression of anti-inflammatory TGF-β and IL-10 (Liu et al., 2019). In addition, BMSCs promoted the M2 microglial polarization in a rat middle cerebral artery occlusion (MCAO) model and suppressed neuroinflammation (Yang et al., 2020). According to some recent studies, MSCs from human exfoliated deciduous teeth reduced neuroinflammation by shifting microglial polarization (M1 to M2) through exosome secretion and paracrine effects (Li et al., 2017; Kitase et al., 2020). In addition to MSCs, transplantation of NSCs showed the function of promoting microglial phenotypic transition from M1 toward M2 phenotype in a study of traumatic brain injury. This study showed the possibility of other stem cells rather than MSCs to induce M2 microglial polarization following SCT (Gao et al., 2016).

Taken together, SCT, especially MSC therapy, are capable of enhancing M2 microglial polarization (Figure 2). The secretion of anti-inflammatory factors by M2 microglia help to alleviate the inflammation and improve the living-conditions of neuronal cells respectively. Related pathways and factors of MSC regulating microglia polarization can be seen in Table 1. Unfortunately, these pathways related to the regulation of microglial polarization have all been identified in studies of the brain rather than the retina. More specific further studies aiming at discovering the effects and related mechanisms of microglial polarization in the retina following SCT are urgently needed.

**TABLE 1:** Related pathways and factors of MSC regulating microglia polarization.

Microglia polarization	Pathways and factors	Species	Stem cell	Model	Paradigm	Results	References
M2-related	TSG-6	Mouse	BMSC	LPS-induced neuroinflammation	*In vivo*	MSC treatment promoted M2 microglial polarization but had little effect with knockdown of TSG-6	Liu et al. (2019)
				LPS-stimulated microglial cell line	*In vitro*		
	MANF	Rat	BMSC	MACO model	*In vivo*	Inhibition of MANF attenuated BMSCs-induced M2 polarization but increased M1 polarization	Yang et al. (2020)
	PDGF-AA			LPS-stimulated microglial cell line	*In vitro*	PDGF-AA treatment enhanced the expression of MANF and increased M2 polarization	
	PI3K/AKT	Rat	BMSC	Deafferentation pain	*In vivo*	BMSC increased M2 polarization and the levels of p-PI3K and p-AKT. However, treatment with PI3K inhibitor impaired BMSC-mediated M2 polarization	Zhong et al. (2020a)
	AMPK	Rat	MSC	SAH model	*In vivo*	MSCs-exosomes promoted the phosphorylation of AMPK and M2 microglial polarization	Han et al. (2021)
M1-related	NF-κB	Rat	BMSC	Deafferentation pain	*In vivo*	BMSC suppressed the NF-κB signaling pathway and M1 polarization	Zhong et al. (2020a)
		Rat	BMSC	SAH model	*In vivo*	MSC-derived exosomes inhibited the NF-κB signaling pathway and M1 polarization	Han et al. (2021)
	CysLT2R	Rat	MSC	MACO model	*In vivo*	MSC-derived exosomes suppressed CysLT_2_R expression and ERK1/2 phosphorylation and inhibited M1 polarization	Zhao et al. (2020)
	ERK1/2						
	C3a-C3aR	Human	UC-MSC	Chronic unpredictable mild stress model	*In vivo*	hUC-MSCs inhibited C3/C3a-C3aR activation signaling and inhibited M1 polarization	Li et al. (2020)

Note: AKT, Protein kinase B; AMPK, AMP-activated protein kinase; BMSC, bone marrow derived mesenchymal stem cells; CysLT2R, CysLT type 2 receptor; C3, Complement C3; ERK, Extracellular signal-regulated kinase; LPS, lipopolysaccharide; MACO, middle cerebral artery occlusion; MANF, Mesencephalic astrocyte-derived neurotrophic factor; MSC, mesenchymal stem cells; PDGF, platelet derived growth factor; PI3K, Phosphatidylinositol 3-kinase; SAH, subarachnoid hemorrhage; TSG-6, Tumor-specific glycoprotein 6; UC-MSC, Umbilical cord-mesenchymal stem cells.

## 6 Future Directions

### 6.1 Cotransplantation of Therapeutic Cells With Mesenchymal Stem Cells-Derived Exosomes May be a Better Solution

**FIGURE 3:**
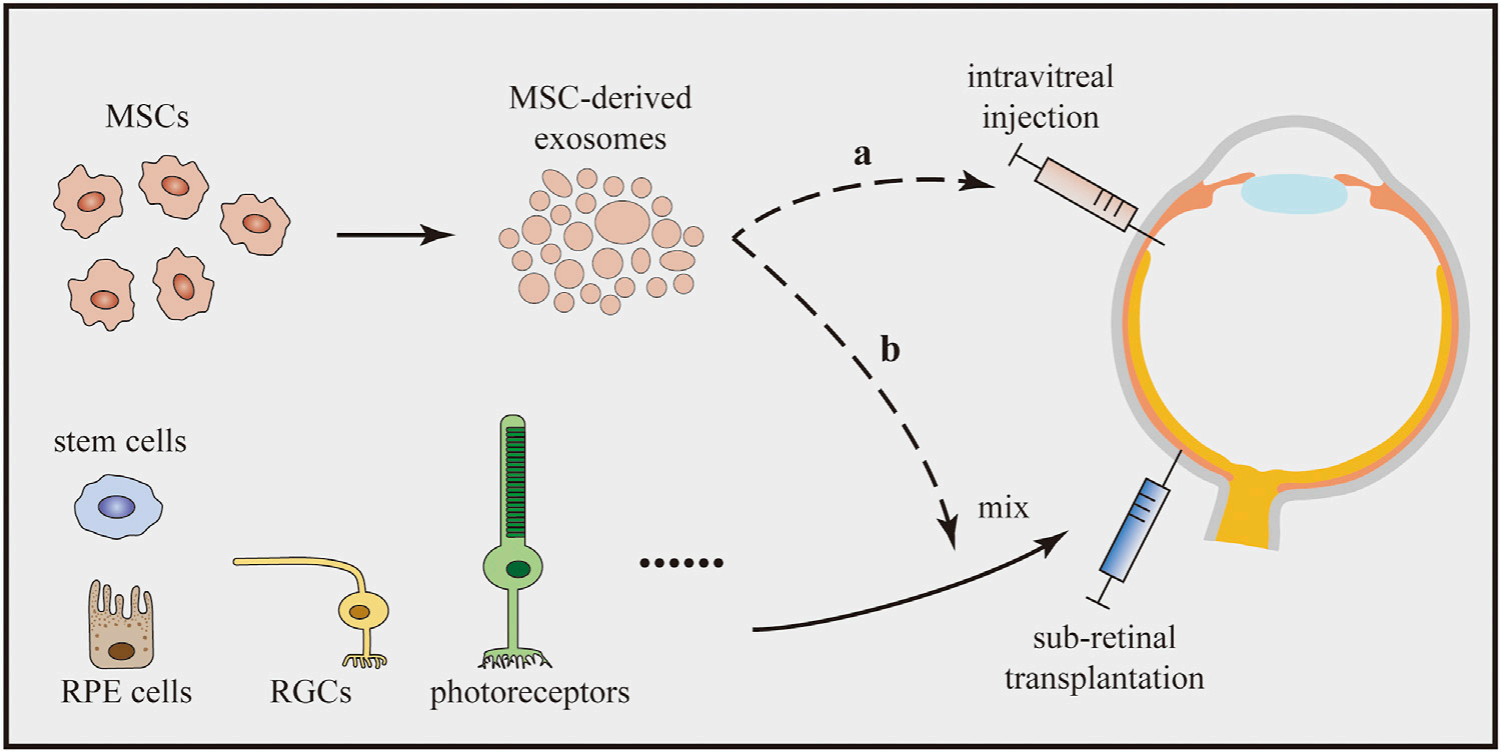
Cotransplantation of therapeutic cells with MSC-derived exosomes. MSC-derived exosomes enhance the M2 microglial polarization and suppress retinal inflammation. The cotransplantation can be implemented by two options: **(a)** The cotransplantation can combine the intravitreal injection of MSC-derived exosomes with subretinal transplantation of therapeutic cells. **(b)** MSC-derived exosomes and therapeutic cells can be mixed in advance and subsequently be transplanted into the subretinal space together.

Injury-induced inflammation is detrimental to the regeneration of retinal cells (Silva et al., 2020). Moreover, the survival and integration of transplanted cells are substantially influenced by microglia and inflammation (Burns and Stevens, 2018). As mentioned above, MSC-derived exosomes are capable of suppressing retinal inflammation by reducing the expression of inflammatory mediators. Additionally, MSC-derived exosomes can enhance the M2 polarization of microglia (Li et al., 2019c). Therefore, we speculate that the cotransplantation of the anticipated cells with MSC-derived exosomes might be a better choice to achieve therapeutic goals (Figure 3). In addition, the application of MSC-derived exosomes remarkably does not cause vitreous opacity, immunologic rejection, or proliferative vitreous retinopathy (Zhang et al., 2018; Mathew et al., 2019). As combined transplantation traditionally requires the injection of at least two cell types, transplantation of a single cell type together with MSC-derived exosomes may decrease the safety risk.

No documents have reported the use of cell-exosome combined therapy to treat retinal diseases. However, recent studies have reported successful paradigms of cotransplantation of exosomes and stem cells and are worth continuing in the field of RDDs. In a rat model of acute ischemic stroke, combined transplantation of ADMSCs and ADMSC-derived exosomes was confirmed to be superior to either ADMSC or ADMSC-derived exosome transplantation alone at reducing the brain infarct zone and improving the recovery of neurological function (Chen et al., 2016). In one study of acute myocardial infarction, the sequential delivery of exosomes and MSCs enhanced the survival of MSCs and reduced their apoptosis both *in vitro* and *in vivo*. Additionally, cardiac function was improved to a greater extent in the cotransplantation group than in the other groups treated with exosomes or MSCs alone (Huang et al., 2019). In addition, Lin et al. demonstrated that the combined treatment of ADMSCs and ADMSC-derived exosomes resulted in the most significant preservation of kidney function and the integrity of kidney architecture compared to the single transplantation groups (Lin et al., 2016).

Regarding the implementation of the new treatment, we assume that two options exist (Figure 3). As MSC-derived exosomes are usually administered by intravitreal injection in studies of eye diseases (Harrell et al., 2018), cotransplantation can combine the intravitreal injection of MSC-derived exosomes and subretinal transplantation of other cells. However, researchers have not clearly determined whether the treatment would work since the two effectors are separately transplanted at different sites. As an alternative, MSC-derived exosomes and other cells could be mixed in advance and subsequently transplanted into the subretinal space. Although these two methods seem to be effective, studies should be carried out to confirm their efficacy. Meanwhile, the underlying cellular interactions and molecular mechanisms also require further research.

### 6.2 Transplantation of Induced Pluripotent Stem Cells-Derived M2 Microglia as a Potential Tool for Retinal Degenerative Disease Treatment

As M2 microglia are more beneficial than M1 microglia in RDDs, the question of whether direct transplantation of M2 microglia into the retina is a feasible approach to modulate the immune microenvironment and alleviate retinal inflammation has been proposed. Unfortunately, no related research has been published yet. However, studies have used this strategy to treat other neurological injuries. Recently, M1 and M2 microglia were transplanted into mouse models of spinal cord injury induced by GM-CSF and IL-4 and marked with CD86 and CD206, respectively. Compared with the control groups, significant recovery was observed in the M2 group, while deterioration was found in the M1 group (Kobashi et al., 2020). Similar outcomes have been reported in studies of brain injuries where transplantation of M2 macrophages improved cognitive impairment in a rat model of AD (Zhu et al., 2016) and transplantation of M2 microglia promoted axonal outgrowth and angiogenesis in a rat model of stroke (Kanazawa et al., 2017).

**FIGURE 4:**
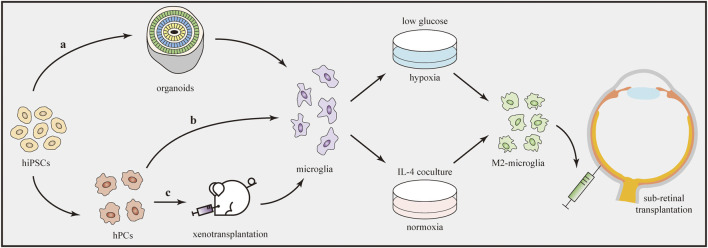
Transplantation of iPSC-derived M2 microglia. Microglia can be generated from hiPSCs through three techniques **(a–c)**. Afterwards, microglia are induced to the M2 phenotype by using oxygen–glucose deprivation technique or IL-4 co-culture treatment. Direct transplantation of M2 microglia into the subretinal space can influence the immune microenvironment and alleviate retinal inflammation.

An obvious limitation is the difficulty of collecting a sufficient number of primary microglia directly from human tissues. Fortunately, recent advances in iPSCs have provided exciting new approaches to overcome this obstacle. Many protocols for generating microglia from hiPSCs have been published (Douvaras et al., 2017; Haenseler et al., 2017; Pandya et al., 2017). Hasselmann and Blurton-Jones (2020) concluded that three techniques are useful to generate iPSC-derived microglia, generally named “*in-vitro* microglia,” “organoid microglia,” and “xenotransplanted microglia” (Figure 4). Each technique features unique routes and has its own benefits and limitations. The “*in-vitro* microglia” technique, as the name implies, differentiates iPSCs into microglia *in vitro*. This technique is superior in terms of high throughput but limited by transcriptomic deficiencies (McQuade et al., 2018). “Organoid microglia” means that microglia are innately generated from iPSC-derived organoids. However, the branching pattern of organoid microglia is still different from that of adult human microglia (Ormel et al., 2018). Two steps are needed to generate “xenotransplanted microglia”. Microglial precursors are first differentiated from iPSCs and then transplanted into the brains of immune-deficient mice carrying the human allele for the colony stimulating factor 1 protein. In one study, microglia produced by this technique showed a better microglial morphology and gene expression signatures that closely resembled those of adult human microglia (Svoboda et al., 2019).

After the production of microglial cells, the next step is to drive them to transform into the M2 phenotype. Several methods have been developed to induce polarization *in vitro* (Figure 4). IL-4 is a cytokine that promotes tissue repair and M2 microglial polarization. According to one study using different concentrations (20 or 40 ng/ml) of IL-4, Arg1 (the M2 marker) expression significantly increased to similar levels in both groups, indicating the dose-independent characteristic of the M2 polarization-stimulating effect of IL-4 (Kobashi et al., 2020). In another study, after preconditioning by oxygen–glucose deprivation, microglia polarized to the M2 phenotype. Under this condition, cultures containing low-glucose medium were first placed in a hypoxia chamber (95% N_2_ + 5% CO_2_) for 1 h and then closed for 18 h (Kanazawa et al., 2017). In addition, several drugs, such as minocycline (Ahmed et al., 2017) and *Lycium barbarum* (Li et al., 2019b), were reported to modulate microglial polarization from the M1 to M2 phenotype in some *in-vivo* models. However, further studies are needed to determine whether they can be used to induce M2 polarization of microglia *in vitro*.

Although current studies are still in their infancy, the development of iPSC-derived microglia has provided insights into the production of abundant M2 microglia for transplantation. As studies in the brain have proven the feasibility and advantages of M2 microglial transplantation, we predict that the transplantation of M2 microglia will also become a proper and effective method to treat RDDs.

## 7 Conclusion

In recent years, SCT in subjects with other neurological disorders has been proven to exert positive effects of switching the polarization of microglia from the M1 to M2 phenotype, providing good examples for the application of SCT in the treatment of RDDs by targeting microglial polarization. However, the function of regulating microglial polarization by SCT in the retina is rarely reported and limited in the field of MSCs. Thus, we wondered if solutions were available to improve this situation and proposed some expectations for SCT to be a better treatment for RDDs, which may be helpful for future research. We postulate that the therapeutic effects of SCT will be improved by cotransplantation therapy with MSC-derived exosomes, which enhance M2 microglial polarization and create an optimized environment for the survival of transplanted cells to achieve better results. Besides, advances in iPSC-derived microglia may promote the development of transplantation of M2 microglia to treat RDDs and alleviate retinal inflammation.
